# Fabrication of Bioprobe Self-Assembled on Au–Te Nanoworm Structure for SERS Biosensor

**DOI:** 10.3390/ma13143234

**Published:** 2020-07-21

**Authors:** Soo Min Kim, Taek Lee, Yeong-Gyu Gil, Ga Hyeon Kim, Chulhwan Park, Hongje Jang, Junhong Min

**Affiliations:** 1 Department of Chemical Engineering, Kwangwoon University, Seoul 01897, Korea; k-soomin@hotmail.com (S.M.K.); 1497rg@hanmail.net (G.H.K.); chpark@kw.ac.kr (C.P.); 2 Department of Chemistry, Kwangwoon University, Wolgye-dong, Nowon-gu, Seoul 01899, Korea; ygland0115@gmail.com; 3 School of Integrative Engineering, Chung-Ang University, Seoul 06974, Korea

**Keywords:** surface-enhanced Raman spectroscopy, aptamer, antibody, Au–Te nanoworm, biosensor

## Abstract

In the present study, we propose a novel biosensor platform using a gold-tellurium (Au–Te) nanoworm structure through surface-enhanced Raman spectroscopy (SERS). Au–Tenanoworm was synthesized by spontaneous galvanic replacement of sacrificial Te nanorods templated with Au (III) cations under ambient conditions. The fabricated Au–Te nanoworm exhibited an interconnected structure of small spherical nanoparticles and was found to be effective at enhancing Raman scattering. The Au–Te nanoworm-immobilized substrate exhibited the ability to detect thyroxine using an aptamer-tagged DNA three-way junction (3WJ) and glycoprotein 120 (GP120) human immunodeficiency virus (HIV) using an antibody. The modified substrates were investigated by scanning electron microscopy and atomic force microscopy (AFM). The optimal Au–Te nanoworm concentration and immobilization time for the thyroxine biosensor platform were further determined by SERS experimentation. Thus, the present study showed that the Au–Te nanoworm structure could be applied to various biosensor platforms.

## 1. Introduction

A biosensor is an analytical device used to precisely detect diseases based on changes in target-receptor signaling or non-target-receptor interaction [1,2]. Several types of biosensors, including electrochemical, electrical, optical, and spectrochemical, have been developed [3,4]. In most cases, biosensors have been devised to improve several functionalities: (1) sensitivity, (2) selectivity, (3) portability, (4) small volume loading, and (5) a user-friendly interface [5,6,7]. Since 2000, advances in nanotechnology have led to the development of biosensors designed to detect various pathogens, including bacteria, viruses, and other microorganisms [8,9,10,11]. By introducing the nanomaterial to the biosensor electrode, the biosensor increased the surface roughness and area, which enhanced the sensitivity [12]. Moreover, some nanomaterials, including quantum dots, upconversion nanoparticles, and graphene, allow the fabrication of new types of fluorescence-based biosensors and magnetic biosensors, among others [13]. In addition, the nanopattern on the biosensor electrode only required a small amount of bioprobe volume, which can reduce the manufacturing cost [14]. Those nanobiosensors have several advantages, such as label-free operation, easy-to-fabricate, and fast response.

Conventional Raman spectroscopy was considered as hard to use in biosensor application due to its weak signal for determining the target. To overcome this problem, the surface-enhanced Raman spectroscopy (SERS) technique can use Raman spectroscopy for biosensor application. In 1974, the first SERS enhancement effect of nanostructure-adsorbed pyridine was reported [15] and there have been reports that the introduction of nanostructures can improve Raman scattering [16,17,18,19,20]. SERS enhancement can be elucidated by electromagnetic field enhancement (EM) and chemical enhancement (CM) [21]. In particular, EM enhancement is regarded as a major contributor to the SERS effect that generates a SERS hotspot between interfaces [22]. SERS-based biosensors have been widely investigated because of their high resolution, molecular fingerprinting ability, non-invasive method, and their support for qualitative analysis of target species.

Recently, various metal nanoparticles with different shapes have provided unique plasmonic behavior to construct SERS-based biosensors [23]. For example, it was reported that through an enhancement of Au nanorods a Raman signal could be elucidated by longitudinal and transverse plasmon bands, an effect which had not been observed in spherical Au nanoparticles [24]. Moreover, it was shown to be the nanospike structure and porous nanostructure that provided this SERS effect [25,26]. Several groups have reported the control of nanoparticle shape for the SERS effect improvement [27,28,29]. Several groups have developed SERS-based biosensors for detecting various pathogen cues, including bacteria, viruses, proteins, and nucleic acids [30,31,32,33]. In the meantime, various Au-based nanocomposite structures have been synthesized for various applications including battery, material, and biomedical applications. Among the applications, Au–Te nanocomposite showed unique characteristics [34,35]. In addition to its unique physicochemical properties, including high surface-to-volume ratio and photo-responsive heat generation, the environmentally friendly synthesis strategy, which does not require the addition of cytotoxic surfactants, can be regarded as a major advantage in various bio-application fields. Moreover, the Au–Te nanoworm structure has a short transverse distance and long longitudinal distance, which can enhance the SERS signal due to the subsequent dual plasmon bands [36,37].

**Figure 1:**
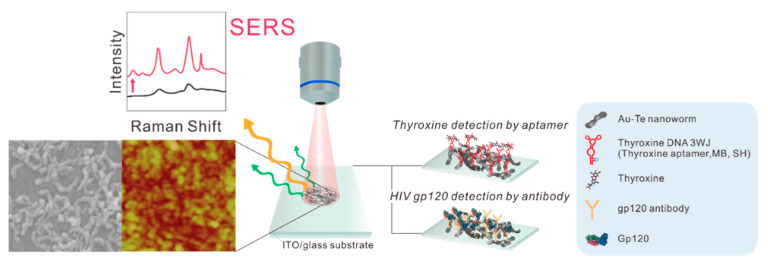
Schematic image of the fabricated biosensor platform. Note: SERS = surface-enhanced Raman spectroscopy, ITO = indium-tin-oxide.

In the present study, we synthesized Au–Te nanoworms and applied them to SERS-based biosensor applications to fabricate aptamer-based biosensors, antibody-based biosensors, and protein-based biosensors. These applications were applied to detect thyroxine (T4), glycoprotein 120 (GP120: the target of human immunodeficiency virus (HIV)), and acute myocardial infarction (AMI) biomarkers, respectively. Preparation of Au–Te nanoworms was accomplished by a spontaneous galvanic replacement reaction of a sacrificial Te nanorod template, as previously reported. The synthesis of the Au–Te nanoworm structure was confirmed by field emission scanning electron microscopy (FE-SEM), ultraviolet-visible spectroscopy (UV-VIS) spectroscopy, and transmission electron microscopy (TEM). The prepared Au–Te nanoworm structure was immobilized on an indium-tin-oxide (ITO) substrate by a chemical linker. Then, each bioprobe, including T4 DNA aptamer, HIV antibody, and myoglobin was self-assembled onto a modified substrate. The fabrication process of each bioprobe-modified Au–Te nanoworm structure was investigated by atomic force microscopy (AFM). The binding events between each target and bioprobe were confirmed by SERS experiments. Figure 1 shows a schematic diagram of the proposed biosensor application.

## 2. Materials and Methods

ITO-glass (3 × 1 cm) was purchased from National Nanofab Center (10 Ω resistance, Daejeon, Korea). L-thyroxine was purchased from Thermo Fisher Scientific (Waltham, MA, USA). GP120 antibody was purchased from Sino Biological (Beijing, China) and GP120 was purchased from ACRO Biosystem (Newark, DE, USA). Triton-X solution, EDC, NHS, and (3-aminopropyl)triethoxysilane (APTES) were purchased from Sigma-Aldrich (St. Louis, MO, USA). Ethylene glycol and sodium hydrogen carbonate were purchased from DaeJung (Siheung-si, Korea). For Au–Te nanoparticle synthesis, poly (vinylpyrrolidone) (Mw = 40 kDa), sodium tellurite, sodium hydroxide, ethylene glycol, hydrazine monohydrate, and chloroauric acid hydrate were purchased from Sigma-Aldrich (St. Louis, MO, USA). All chemicals were used as received. The sequences of the strands used as thyroxine probes were as follows: the aptamer of thyroxine used to create the DNA 3WJ-a strand was 5′-TAA TAC GAC TCA CTA TAG GGA ATT CGT CGA CGG ATC CGC CGT TGG TGT TCG GTC AGG CTT CCG TGG CAA CGG GGC AAA ATG GTA ATC GCG GGG AAC CCT GCA GGT CGA CGC ATG CGC CGT TGC CAT GTG TAT GTG GG-3′ and was synthesized by IDT (Coralville, IA, USA); DNA 3WJ-b modified methylene blue was MB-5′-CCC ACA TAC TTT GTT GAT CC-3′; and DNA 3WJ-c modified thiol was SH-5′-GGA TCA ATC ATG GCA A-3′. DNA 3WJ-b and DNA 3WJ-c were synthesized and purified using HPLC by Bioneer (Daejeon, Korea). The ultrapure water used in the experiment was purified by VENUS ROUP-15-S from Jeongbiotech (Incheon, Korea).

### 2.1. Preparation of Au–Te Nanoworms

Au–Te nanoworms were synthesized by galvanic replacement of sacrificial Te nanorods under aqueous dispersion conditions [38]. Briefly, Te nanorods were first prepared by seed-mediated growth in ethylene glycol solvent. More specifically, 1 g of poly(vinylpyrrolidone) (PVP, Mw = 40 kDa), 92.2 mg of sodium telluride, and 0.5 mg of NaOH were fully dissolved in 40 mL of ethylene glycol by vortexing. Ultrasonication was not applied to avoid nucleation; 1.3 mL of hydrazine monohydrate was added, followed by heating for 3 h at 70 °C with vigorous magnetic stirring. The synthesized Te nanorods were purified by dialysis using a 30 kDa cutoff membrane in a distilled water reservoir.

A quantity of 2.5 mL of 10 mM HAuCl4 aqueous stock solution was rapidly injected to the 20 mL of as-prepared Te nanorods, followed by 2 h of incubation at room temperature to achieve galvanic replacement and nanoworm formation. Manufactured Au–Te nanoworms were purified by centrifugation (8000 rpm for 10 min) and further washed three times with distilled water to confirm the removal of unreacted and dissolved ions. Finally, the Au–Te nanoworms were re-dispersed in 20 mL of distilled water for further use.

### 2.2. Immobilization of Au–Te on ITO

The ITO substrate was treated with atmospheric plasma for 5 min to form a hydrophilic surface. Subsequently, 30 µL of 5% APTES was covered for 12 min on the surface for silanization, rinsed in ethanol, and dried under a stream of nitrogen gas. After drying, the substrate was heat-treated at 70 °C for 1 h to prepare an amine-modified SAM. Thereafter, 40 µL of Au–Te nanoparticles were dropped on the surface of the ITO, which was then incubated for 3 h at room temperature to prepare Au–Te/ITO substrates through self-assembly of amine groups and Au. Finally, unbound Au–Te was washed with deionized water (DIW) and dried under a nitrogen gas stream.

### 2.3. Fabrication of Target/Biomolecules/Au–Te on ITO

In this experiment, a biosensor of thyroxine and GP120 was prepared. First, the DNA 3WJ structure was used as the thyroxine detection probe. Each of the three DNA strands is functional and assembled to form a single nanostructure. The DNA 3WJ-a strand can capture thyroxine, and the DNA 3WJ-b strand was labeled with methylene blue to confirm the Raman signal of the DNA probe. Strand c labeled thiols and formed covalent bonds with Au–Te particles, allowing the DNA 3WJ probe to be immobilized on the substrate. To assemble each of these DNA fragments, they underwent 5 min of heat treatment at 80 °C in the TMS buffer and were then cooled at 4 °C for 30 min; 30 µL of the assembled DNA 3WJ probe solution was placed on an Au–Te/ITO glass substrate, incubated at room temperature overnight, washed with distilled water, and dried under nitrogen gas to prepare the DNA 3WJ/Au–Te/ITO substrate. To detect thyroxine, 30 μL of 1 mM thyroxine diluted in dilution buffer (50% ethylene glycol, 500 mM NaHCO_3_) was dropped on the substrate and immobilized at room temperature for 3 h.

For immobilization of GP120 antibodies on Au–Te/ITO, 100 μL of the GP120 antibody (5 μg/mL) was mixed with 100 μL of EDC (4 mg/mL) and NHS (6 mg/mL) for 1 h at room temperature. The Au–Te/ITO electrode was treated with cysteamine; 10 μL of 10 mM cysteamine (dissolved in ethanol) was dropped onto the Au–Te/ITO substrate to form a self-assembled thin film. Then, the unbound cysteamine was removed by washing under DIW and N_2_ gas. Thirty microliters of the EDC/NHS-treated antibody solution was dropped on the cysteamine-treated Au–Te/ITO and allowed to react overnight at room temperature. All samples were washed with deionized water and dried under a nitrogen gas stream before measurement.

### 2.4. Surface Morphology Analysis

The surface of the Au–Te/ITO substrate was confirmed by FE-SEM (Auriga, Carl Zeiss, Germany) and compared with the AFM (Digital Instruments, Billerica, MA, USA) results. Au–Te/ITO substrates were investigated using the tapping mode AFM, using phosphorous (n-type doped Si, RTESP, Bruker, Billerica, MA, USA) tips. The integral gain, proportional gain, and setpoint current were optimized for the force between the tip and the substrate surface before scanning the sample.

### 2.5. Measurement of Surface-Enhanced Raman Scattering

SERS measurements were performed for DNA 3WJ aptamer-thyroxine binding, GP120 antibody GP120 protein binding, and myoglobin. SERS was measured using a SENTERRA confocal Raman spectroscope (Bruker Optics, Billerica, MA, USA) using a 785 nm diode laser with 10 mW power; an exposure time of 5 s was set for all experiments. Raman spectra were obtained using the average results from 10 different points in 10 independent samples.

## 3. Results

### 3.1. Investigation of Immobilized Biomolecules/Au–Te on the ITO Substrate

**Figure 2:**
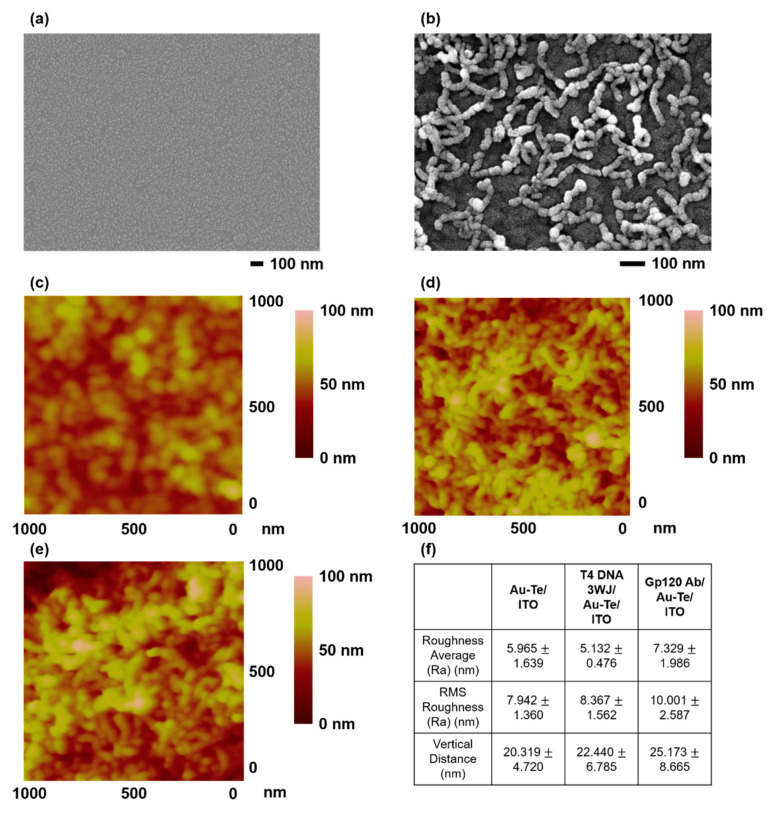
Field emission scanning electron microscopy (FE-SEM) image of (**a**) Bare indium-tin-oxide (ITO), (**b**) Au–Teon ITO. Atomic force microscopy (AFM) image of (**c**) Au–Te/ITO, (**d**) T4 DNA 3WJ/Au–Te/ITO, (**e**) GP120 antibody/Au–Te/ITO. (**f**) Surface roughness analysis of the Au–Te/ITO, T4 DNA 3WJ/Au–Te/ITO, and GP120 Ab/Au–Te/ITO (The surface roughness analysis was carried out at 10 different positions on the AFM images, respectively).

The surface of the ITO substrate immobilized with biomolecules/Au–Te was investigated by AFM and FE-SEM. Figure 2a,b shows the FE-SEM results before and after immobilization of Au–Te nanoworms structure on an ITO substrate. Figure 2b shows that the earthworm-shaped Au–Te nanoworms structure was well immobilized on ITO. The Au–Te nanoworms structure immobilized on the ITO substrate were approximately 200–220 nm in length and 20 nm in height. Figure 2d,e shows the AFM results after immobilization of T4 DNA 3WJ and GP120 antibody on Au–Te/ITO substrate. An increase of approximately 2–3 nm in height was measured on substrates immobilized with T4 DNA 3WJ (Figure 2d). Figure 2e shows that the total vertical height increased by 5 nm due to the addition of the GP120 antibody to the Au–Te particles immobilized with EDC/NHS. Compared to the Au–Te nanoworms structure, the surface morphology and surface roughness analysis showed different shapes and values. When the biomolecules (T4 DNA 3WJ and GP120 antibody) were added on the Au–Te nanoworm-modified substrate, respectively, we found some of the values and morphology changed. Presumably, the interaction between some of biomolecule and nanostructure gives the difference of values. Moreover, the increment of vertical distance can be interpreted to be stacking of biomolecules on the Au–Te nanowrom structure. The overall result confirmed that the biomolecule/Au–Te layer is well immobilized on the ITO substrate.

### 3.2. Enhancement Factor of Au–Tenanoworms

**Figure 3:**
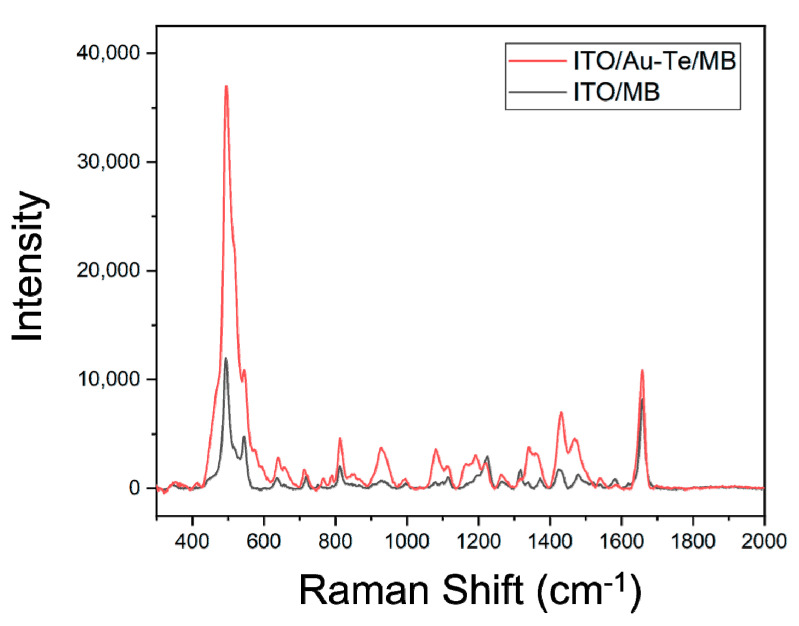
Raman spectrum of methylene blue on ITO, ITO/Au–Te.

**Figure A1:**
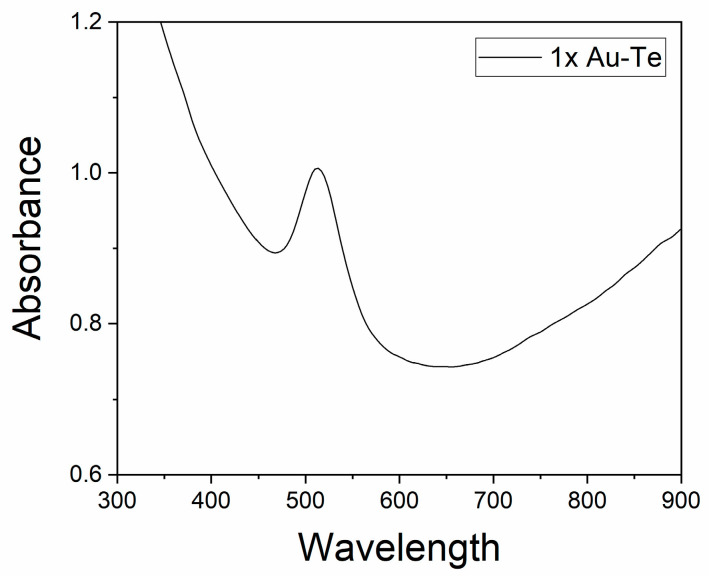
Absorbance of 1× Au–Te.

To calculate the EF of the Au–Te nanoworm, the ratio of SERS to the normal Raman spectrum (NRS) of methylene blue was determined using the following equation [39,40]. The laser was irradiated at 785 nm and compared to the intensity at 495 cm^−1^, where the methylene blue peak was apparent as shown in Figure 3. The Raman intensity was compared by measuring a sample in which 40 µL of 1 mM methylene blue was dropped on the cleaned ITO and a sample of the same amount of methylene blue on ITO with 1× concentration of Au–Te nanoparticles (1× reference absorbance Figure A1).

(1) $$\mathrm{EF}=\left(I_{\mathrm{SERS}}/N_{\mathrm{SERS}}/I_{\mathrm{NRS}}/N_{\mathrm{NRS}}\right)$$

(2) $$N_{\mathrm{SERS}}=N_{A}{\mathrm{nS}}_{\mathrm{Irr}}/S_{\mathrm{dif}}$$

(3) $$N_{\mathrm{NRS}}={\mathrm{dN}}_{A}{\mathrm{hS}}_{\mathrm{Irr}}/M$$

$I_{\mathrm{SERS}}$ and $N_{\mathrm{SERS}}$ are the intensity of SERS and NRS, respectively, and the ratio is calculated as 3.2. $N_{A}$ is the Avogadro’s constant, n is the number of moles of the molecule, $S_{\mathrm{Irr}}$ is the irradiation region under the laser beam (1.5 μm in diameter), and $S_{\mathrm{dif}}$ is the diffusion region of the sample material to be tested. In the test, 1 mM of methylene blue 40 μL solution was dropped on an Au–Te nanoworm substrate, dried, and a circle having a diameter of 3 mm was formed. Therefore, about 1.5 × 10^−9^ molecules were present in the laser beam spot ($N_{\mathrm{SERS}}$).

The term d is the packing density of methylene blue, the size of methylene blue is about 14 × 9 Å, but it is assumed to be a spherical molecule having a diameter of 10 Å, and it is calculated assuming that it is composed of a monolayer, and the result is 1.1954 × 10^−15^ g/1.178 × 10^−3^ μm^3^. The term h refers to the laser confocal depth (2.8 μm) and M corresponds to the molecule weight of methylene blue. Therefore, about 3 × 10^−9^ molecules were present in the laser beam spot ($N_{\mathrm{NRS}}$). Finally, the EF value is 6.4.

This shows that the EF of the Au–Te based SERS application is significantly lower compared to the fact that the EF of the SERS applications using gold or silver is 10^6^–10^11^, but the small molecule thyroxine can be detected without labeling [41,42]. In addition, it is considered to be utilitarian when introducing biosensors because it is possible to synthesize large quantities of nanoparticles that can enhance Raman signals without using precious metal nanoparticles.

### 3.3. Optimization of Au–Te Immobilization on ITO

**Figure 4:**
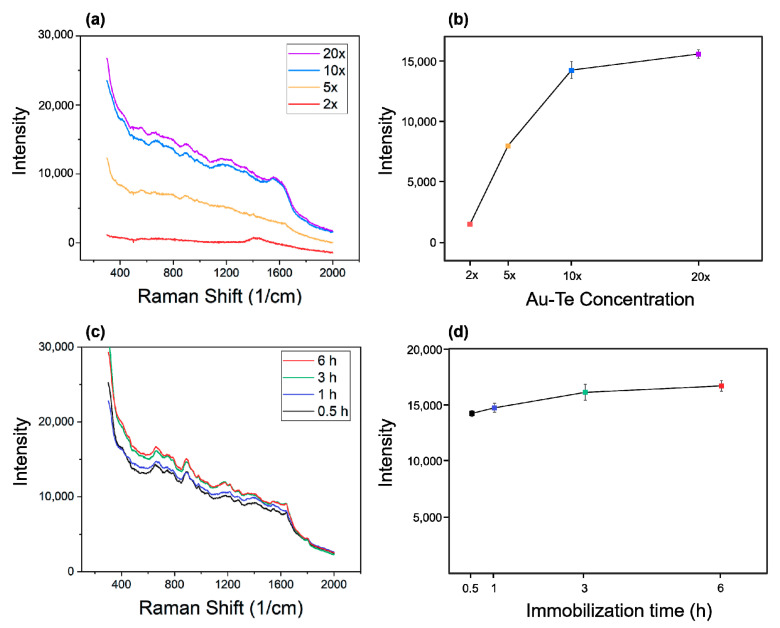
Optimum conditions for Au–Te immobilization on ITO. (**a**) Raman spectra of various Au–Te concentrations on an ITO substrate, (**b**) the SERS effect of Au–Te concentrations, (**c**) Raman spectra with different immobilization time conditions of Au–Te on ITO, (**d**) the SERS effect of Au–Te immobilization time.

In order to maximize the Raman enhancement effect of Au–Te particles, we monitored the change in Raman intensity by varying the concentration and immobilization time, and set the optimal concentration and immobilization time accordingly. Figure 4a shows the Raman spectra at various concentrations of Au–Te (1× reference absorbance Figure A1). The initial 2× spectra were the Raman spectra of ITO itself, indicating presence of few Au–Te particles. The Raman intensity was observed to increase with increasing Au–Te concentration (Figure 4b). The plot of the Raman spectrum of Au–Te 10× particles with immobilization time is shown. The Raman intensity gradually increased with immobilization time. However, after 3 h, the spectrum was almost the same. As a result, we adopted a 10× concentration of Au–Te and a 3 h immobilization, as this showed the greatest efficiency.

### 3.4. Detection of Biomolecules by SERS

Before confirming the binding peak between DNA 3WJ and T4, the Raman peak of T4 enhanced by Au–Te nanoworm was identified. The enhanced Raman scattering peak of T4 was found to be very high at 783, 891, 974, 1087 and 1615 cm^−1^. Table 1 shows the vibration allocation for the Raman peak of T4 [43]. The peaks of T4 DNA 3WJ were found to be 501, 780, 1360 and 1656 cm^−1^. The peak at 501 cm^−1^ was typical of methylene and was confirmed to be due to the methylene blue modification of the T4 DNA 3WJ strand [44]. The peak at 780 cm^−1^ was confirmed by the peak of the DNA phosphate band and thyroxine cytosine [45]. To confirm the detection performance of T4, 3 mM T4 was dropped on a T4 DNA 3WJ/Au–Te ITO substrate, and then SERS measurement was performed. As a result, peaks of 495, 779, 872, 896, 1092, 1267, 1607 and 1656 cm^−1^ were observed after T4 binding to T4 DNA 3WJ. The peaks of 779, 896, 1092, and 1607 cm^−1^ are believed to be the result of the presence of T4. The frequencies of 779 and 896 cm^−1^ are common frequencies that appeared in both T4 and T4 DNA 3WJ, but the intensity was much increased, and Raman signals in the 1092 and 1607 cm^−1^ regions, which were not present in T4 DNA 3WJ, were detected. However, the frequency shift appears to have occurred at 1087 and 1615 cm^−1^, which was the peak of the original T4. In addition, it was confirmed that the Raman signal of 495 and 1656 cm^−1^ frequency identified as the peak of T4 DNA 3WJ was present, indicating that T4 DNA 3WJ was also present on the substrate and that T4 and T4 DNA 3WJ were detected together. These results confirmed that T4 was detected using T4 DNA 3WJ.

**Figure 5:**
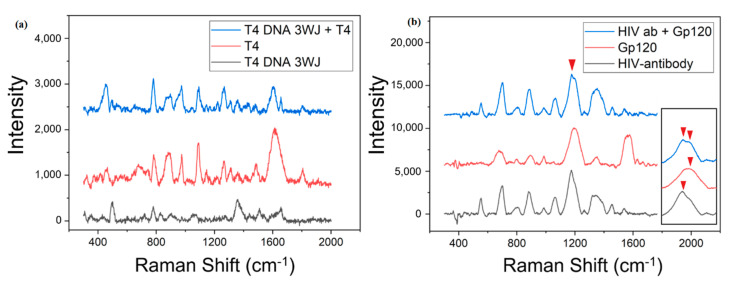
(**a**) Detection of thyroxine on DNA 3WJ/Au–Te/ITO by SERS, (**b**) detection of GP120 on HIV-antibody/Au–Te/ITO by SERS.

The second Raman spectrum that detected GP120 is shown in Figure 5b. After measuring the Raman spectra of the GP120 antibody and GP120 individually, we compared the results after the binding of GP120 to the GP120 antibody. The most prominent frequency of the HIV antibody was 1177 cm^−1^, and the highest peak was found at 1196 cm^−1^ in GP120. Looking at the enlarged spectrum at 1200 cm^−1^ on the right side of Figure 5b, there are 1177 and 1199 cm^−1^ peaks in the Raman spectrum in which the HIV antibody and GP120 are bound, and a slight frequency shift occurs. In addition, a high peak in the region of 1341 cm^−1^ in the HIV antibody and a low intensity in 1354 cm^−1^ in GP120 were observed. In the antibody–antigen conjugation, peaks of 1341 and 1354 cm^−1^ were both confirmed and intensity increased. Lastly, in the 1561–1576 cm^−1^ region a prominent peak in GP120 disappeared after binding with the antibody. It has been reported that frequency shifting in SERS-based immunoassays may be due to structural modifications that occur during antibody–antigen conjugation [46,47]. In addition, there have been no reports of the extinction of the Raman signal in the immunoassay method, but there are reports of the extinction of the Raman signal at the single-molecule level [48]. As a result, antigen antibody conjugation without labels could be confirmed by SERS. Each peak assignment of GP120 is shown in Table 2 [49,50].

Compare this study with other SERS-based sandwich immunoassays, the experimental methods label the antibody to cause signal amplification, but it is inconvenient to add further labeling to the detector antibody, the entire protocol is very long, and only the antigen–binding antibody has to be separated [51]. The disadvantage is that it takes much additional time to detect pathogenic substances that need to be detected quickly, such as viruses, and thereby one cannot provide rapid results to patients and medical staff. However, in this study, it can be said that the capture antibody is fixed on the substrate without labeling, and after binding with the antigen, the unbound sample can be easily separated by washing, and it is easy to detect quickly due to the short production step. As a result, this study is a SERS platform that can quickly detect the antigen without labeling of target substances with only the augmented Raman signal of Au–Te nanoparticles. We believe this platform will be easier to apply in the field where rapid detection is needed.

## 4. Conclusions

In this study, authors developed a SERS biosensor platform using Au–Te nanoworm particles. For validating the biosensor platform, we introduced two types of bioprobes. The first bioprobe is the aptamer for T4 recognition, and the second bioprobe is the antigen–antibody immune response sensor for detecting HIV via the target GP120 protein. The aptamer peak was clearly identified by labeling the aptamer with methylene blue, and the peak of the target molecule was observed even after binding to the target molecule. Antigen–antibody immune response sensors were able to detect the presence of antigen through peak analysis without labeling. The EF of the Au–Te based SERS application showed a low level of 3.2, but showed that a single molecule level can be detected without labeling. In addition, Au-based nanostructures formed by galvanic replacement with high-complexity have low yields. This is because noble metal nanoparticles such as Ag and Cu used as sacrificial nanotemplates are difficult to synthesize in large quantities. However, when Te, a typical element, is used, the yield of the template is improved by about 100 times or more, so it can be said that it has an advantage in practical use. As a result, it was confirmed that it has a high potential for application to SERS-based biosensors because it measures target materials without labels and has the advantages of fast detection time and small samples. In the near future, Au–Te nanoworm can be used as the powerful candidate for SERS biosensor construction.

## Figures and Tables

**Table 1:** Assignments of SERS peak of Thyroxine.

Wave Number (cm^−1^)	Vibrational Assignments
783	υ(C^3^—O)10, υ(C^32^—C^27^)10, β(C^2^—C^1^—C^6^), β(C^4^—C^5^—C^6^)10, β(C^5^—C^6^—O^17^)13
891	τ(H^15^—C^10^—C^11^—C^12^)48
974	υ(C^27^—N), y(C^24^—C^27^)17, β(H^30^—N^29^—C^27^)20, τ(H^7^—C^1^—C^2^—C^3^)10
1087	υ(C^9^—C^24^)11, β(H^16^—C^14^—C^13^)45
1615	β(N^29^H^2^)84, τ(H^30^—N^29^—C^27^—C^24^), τ(H^31^—N^29^—C^27^—C^24^)13

**Table 2:** Assignments of SERS peak of GP120.

GP120	
Wave Number (cm^−1^)	Assignment
675	D-Mannose
798	C, U, Thr
891	β-C1 config. Trp, Val Man
987	Man, ribose
1200	Tyr, Phe
1354	Trp
1561	GlcNac(Amid II) Amide II, Trp
1576	G, A, Trp

## Appendix A

The synthesized Au–Te nanoworm particle solution was based on a concentration of 1× when absorbance was 1. The absorbance graph is shown below (Figure A1).
